# Increased FLYWCH1 Expression is Negatively Correlated with Wnt/β-catenin Target Gene Expression in Acute Myeloid Leukemia Cells

**DOI:** 10.3390/ijms20112739

**Published:** 2019-06-04

**Authors:** Amany Almars, Panagiota S. Chondrou, Emenike K. Onyido, Sheema Almozyan, Claire Seedhouse, Roya Babaei-Jadidi, Abdolrahman S. Nateri

**Affiliations:** 1 Cancer Genetics & Stem Cell Group, Cancer Biology, Division of Cancer and Stem Cells, School of Medicine, University of Nottingham, Nottingham NG7 2UH, UK; ttxaial@exmail.nottingham.ac.uk (A.A.); msxpc4@nottingham.ac.uk (P.S.C.); e.k.onyido@swansea.ac.uk (E.K.O.); ttxsa97@exmail.nottingham.ac.uk (S.A.); mszrb3@exmail.nottingham.ac.uk (R.B.-J.); 2 Haematology, Nottingham City Hospital, Division of Cancer and Stem Cells, School of Medicine, University of Nottingham, Nottingham NG5 1PB, UK; mrzcs@exmail.nottingham.ac.uk; 3 Respiratory Medicine, School of Medicine, University of Nottingham, Nottingham NG7 2UH, UK

**Keywords:** acute myeloid leukaemia (AML), leukemic stem cell (LSC), FLYWCH1, WNT/β-catenin

## Abstract

Acute myeloid leukaemia (AML) is a heterogeneous clonal malignancy of hematopoietic progenitor cells. The Wnt pathway and its downstream targets are tightly regulated by β-catenin. We recently discovered a new protein, FLYWCH1, which can directly bind nuclear β-catenin. Herein, we studied the FLYWCH1/β-catenin pathway in AML cells using qRT-PCR, Western blot, and immunofluorescence assays. In addition, the stemness activity and cell cycle were analysed by the colony-forming unit (CFU) using methylcellulose-based and Propidium iodide/flow cytometry assays. We found that FLYWCH1 mRNA and protein were differentially expressed in the AML cell lines. C-Myc, cyclin D1, and c-Jun expression decreased in the presence of higher FLYWCH1 expression, and vice versa. There appeared to be the loss of FLYWCH1 expression in dividing cells. The sub-G0 phase was prolonged and shortened in the low and high FLYWCH1 expression cell lines, respectively. The G0/G1 arrest correlated with FLYWCH1-expression, and these cell lines also formed colonies, whereas the low FLYWCH1 expression cell lines could not. Thus, FLYWCH1 functions as a negative regulator of the Wnt/β-catenin pathway in AML.

## 1. Introduction

Acute myeloid leukaemia (AML) is a malignancy characterized by uncontrolled proliferation and failure in stem cell lineage differentiation, causing the accumulation of non-functional cells called myeloblasts [1]. AML has an incidence of 3.7 per 100,000 people in Europe and is the most frequent malignant myeloid disease in adults (>65 years age group) [2,3]. Diverse genetic aberrations result in the heterogeneity of the disease and lead to differing responses to chemotherapies [4]. Leukaemia stem cells (LSCs), a small subpopulation of cells that often survive therapy, are a recognised cause of relapse. LSCs were recently shown to share many properties of normal hematopoietic stem cells (HSC), including self-renewal, quiescence, and multipotency, and also of some molecular machinery. 

The Wnt/β-catenin pathway plays a critical role in hematopoietic stem cell (HSC) self-renewal, and its impairment might also affect LSCs and AML cells. Wnt pathway activation leads to the accumulation of β-catenin in the cytoplasm and its translocation to the nucleus, where it replaces the Groucho proteins [5]. It interacts with T-cell factor and lymphoid enhancer factor, promoting the transcription of downstream genes such as c-*MYC*, c-*JUN*, and cyclin D1 (*CCND1*) [6]. β-catenin upregulation impairs multi-lineage differentiation and impairs the HSC pool [7,8,9], and is required for the development of LSCs in AML [10]. Recently, it was also demonstrated that deregulation of Wnt/β-catenin pathway resulted in different malignancies, including AML [11,12,13,14]. Although the mechanism of action is still not clearly understood, tight regulation of the β-catenin pathway appears to be a requirement for maintaining the stem cell phenotype [10,15]. Given the regulatory importance of nuclear co-factors (both co-activators and co-repressors) on the Wnt signalling pathway, it is possible that any newly described nuclear β-catenin partner could have crucial involvement in normal versus leukaemia cell development.

Identifying proteins that regulate nuclear β-catenin, particularly in AML, is necessary for controlling the aberrant Wnt/β-catenin pathway. Apart from TCF4, the involvement of several other DNA-binding transcription factors with β-catenin is reported in the literature [16,17,18,19]. To further delineate the nuclear events of the Wnt signalling pathway, we used a modified yeast two-hybrid Ras-Recruitment System (RRS) [20,21], using mouse embryonic-cDNA library and identified several new proteins that bind β-catenin in a GSK-3β phosphorylation-dependent and/or independent manner [22]. One of the β-catenin interacting protein was FLYWCH1, a conserved nuclear protein with multiple FLYWCH-type zinc-finger domains [22]. FLYWCH1 is a previously uncharacterized protein product of the human *FLYWCH1* gene. Interestingly, the majority of identified transcription regulators associated with β-catenin, for example; KLF4 [23], and Glis2 [24] belong to the Zinc Finger Protein (ZFP) family, which is characterized by having multiple Cys2-His2 (C2H2)-type zinc-finger DNA-binding domains [25,26]. The FLYWCH motif has recently been determined and isolated from the *Drosophila* modifier of the mdg4 protein [27,28]. In addition, FLYWCH motifs were also identified and studied in two more proteins of *Caenorhabditis elegans*: PEB-1 [29] and FLYWCH transcription factors; FLH-1, FLH-2, and FLH-3 [30]. The human *FLYWCH1* gene has been mapped on chromosome 16, and contains five FLYWCH-type zinc finger motifs that are highly conserved between mammals. The role of FLYWCH is unclear; however, based on our recent study, FLYWCH1 antagonizes β-catenin/TCF4 signalling during cell polarity/migration in colorectal cancer [22]. Accordingly, the available literature for the role of mammalian FLYWCH1 is limited. Therefore, we decided to study FLYWCH1 in association with β-catenin in AML and hypothesized that it regulates nuclear β-catenin activity in AML cells. This study uncovers a new molecular mechanism by which FLYWCH1, with a possible tumour suppressive role, represses nuclear β-catenin activity in AML cell lines. 

## 2. Results

### 2.1. FLYWCH1 mRNA was Differentially Expressed in the AML Cell Lines

**Figure 1:**
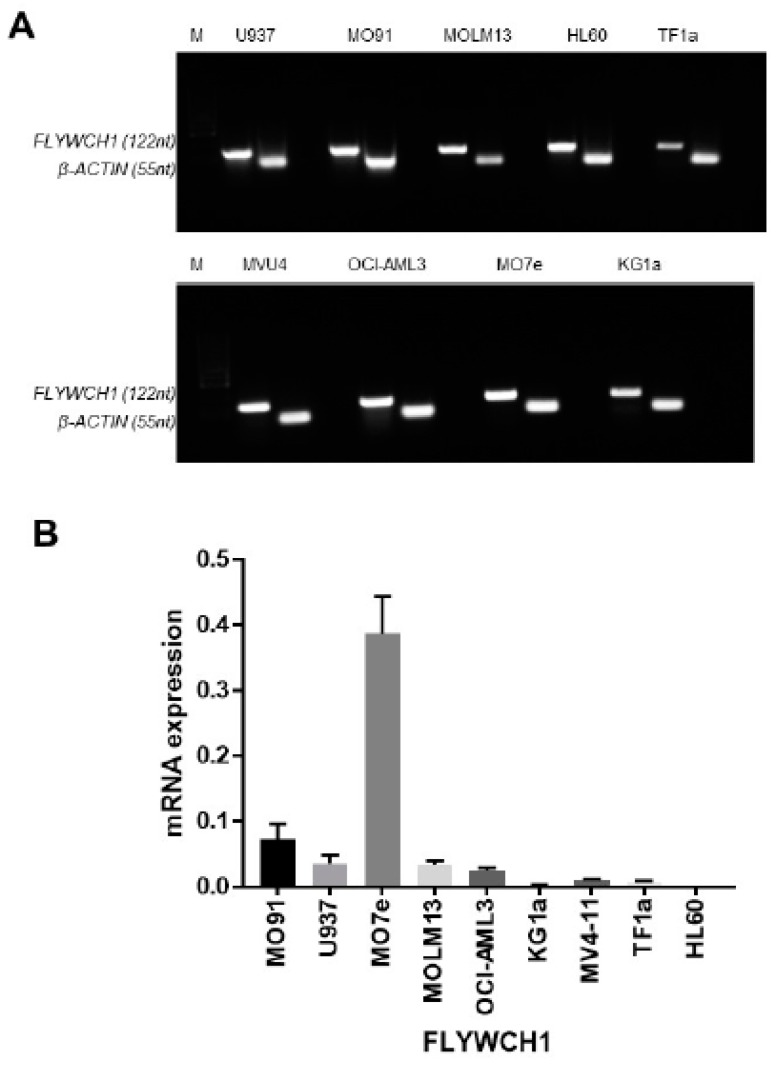
*FLYWCH1* mRNA is differentially expressed in acute myeloid leukaemia (AML) cells. (**A**) RT-PCR of *FLYWCH1* (129 bp) in AML cell lines; β-actin (55 bp) was the housekeeping gene. The samples were electrophoresed in an ethidium bromide–stained agarose gel. (**B**) qRT-PCR analysis of *FLYWCH1* mRNA expression in AML cell lines. Data were normalized to β-actin according to its threshold cycle values, where the comparative threshold cycle value (2^−ΔCt^) was calculated. Error bars show the SD of triplicates samples, from 3 independent measurements.

We initially examined the overall level of *FLYWCH1* mRNA expression and the specificity of designed primers, in nine AML cell lines using RT-PCR. The RT-PCR data confirmed that *FLYWCH1* primers render a single product (Figure 1A). An overall differential expression of *FLYWCH1* in the AML cell lines were apparent using RT-PCR (Figure 1A). Next, we performed real time quantitative PCR (qRT-PCR). The qRT-PCR investigation of *FLYWCH1* mRNA expression confirmed the differential expression of *FLYWCH1* mRNA among the cell lines. *FLYWCH1* mRNA expression was highest in the M07e cell line, followed by that in the M091, MOLM-13, U937, and OCI-AML3 cell lines. *FLYWCH1* mRNA expression was lowest in the KG1a, TF-1a, and HL-60 cell lines (Figure 1B).

### 2.2. Immunofluorescence Staining Indicated Differential FLYWCH1 Protein Expression Levels in AML Cell Lines 

**Figure 2:**
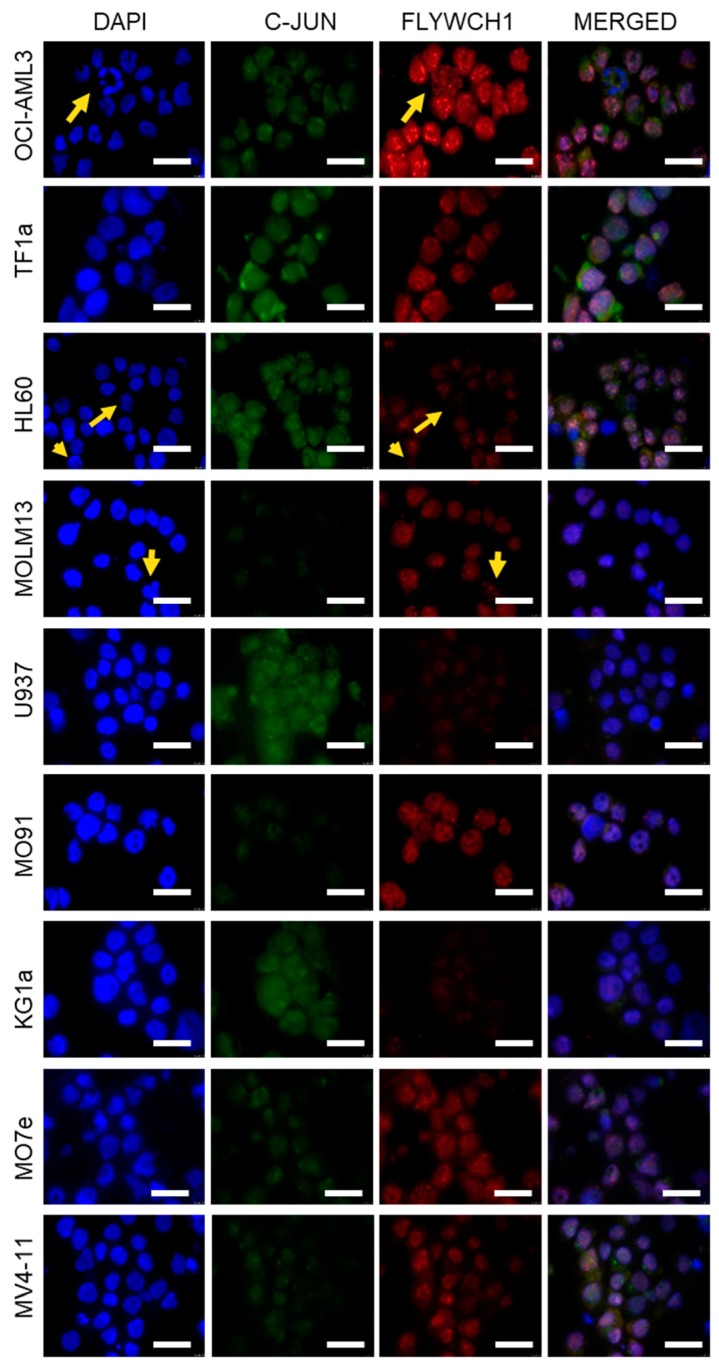
Diverse expression of FLYWCH1 and c-JUN proteins in AML cell lines. Immunofluorescence (IF) analysis of FLYWCH1 (red), c-JUN (green), with DAPI nuclear counterstain (blue). The loss of FLYWCH1 expression in dividing cells is demonstrated in magnified insets (yellow arrowheads). Scale bars, 50 μm.

So far, there is no commercial antibody against FLYWCH1 for western blot detection of endogenous FLYWCH1. Therefore, we examined FLYWCH1 protein expression levels with immunofluorescence staining (Supplementary Figures S1 and S2, red panels). Cells were stained with anti-FLYWCH1, anti-c-JUN antibodies, and DAPI DNA staining. FLYWCH1 was observed as nuclear punctate staining in the AML cell lines. There was differential expression of FLYWCH1 protein in the cell lines (Figure 2). The fluorescence imaging indicated that FLYWCH1 protein expression was highest in the MV4-11, OCI-AML3 and HL-60 cell lines, while expression was moderate in the MOLM-13, M091, TF-1a, and M07e cell lines. FLYWCH1 protein expression was lowest in the KG1a and U937 cell lines. However, the level of mRNA in some of the cells, for example, M07e, was not directly correlated with protein expression (Figure 1B versus Figure 2, red panels), presumably due to post-transcriptional and posttranslational processes. Interestingly, we also noted that dividing cells seemed to lose FLYWCH1 expression. Dividing cells in the M07e, OCI-AML3, KG1a, and MV4-11 cell lines showed loss of FLYWCH1 expression (Figure 2, yellow arrowheads). 

### 2.3. FLYWCH1 Represses the Expression of the β-catenin Target Genes

**Figure 3:**
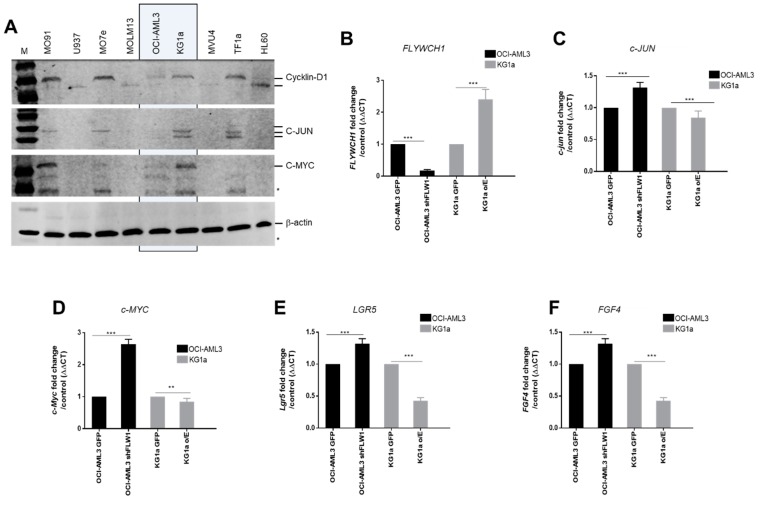
FLYWCH1 regulates certain β-catenin/TCF4 target genes involved in the AML. (**A**) Western blot analysis of β-catenin target gene expression in AML cell lines using antibodies against cyclin D1, c-Myc, c-Jun, and β-actin loading control. Asterisks (∗) indicate unspecific bands. (**B**–**F**) Effect of (**B**) *FLYWCH1* gene expression modulation on: (**C**) *c-JUN*, (**D**) *c-MYC*, (**E**) *LGR5*, and (**F**) *FGF4* transcription activity. Data are mean ± SD (*n* = 6; * *p* < 0.05; ** *p* < 0.01; *** *p* < 0.001).

Western blotting of c-Myc, cyclin D1, and c-Jun was performed to evaluate whether FLYWCH1 is associated with the putative downstream targets of the Wnt/β-catenin pathway (Figure 3A). The results suggested that the expression of the downstream targets was repressed in the AML cell lines with higher FLYWCH1 protein expression, i.e., M07e, OCI-AML3, and HL-60. By contrast, c-Myc, cyclin D1, and c-Jun were expressed at higher levels in the cell lines with low FLYWCH1 expression, i.e., KG1a. Consistent with this observation, *FLYWCH1*-knockdown in OCI-AML3 (Supplementary Figure S2A), significantly induced the expression of β-catenin target genes, e.g., c-Myc [31], FGF4 [32], PPARD [33], CXXC4 [34], LGR5, and c-Jun (Figure 3B–F), but expression was repressed by FLYWCH1-overexpression in KG1a cells (Supplementary Figure S2B and Figure 3B–F). These results demonstrated that FLYWCH1 antagonizes the β-catenin-induced gene expression in AML cells. 

### 2.4. Significant Differences between sub-G0 and G0/G1 Phases of Low and High FLYWCH1 Expression Cell Lines

To evaluate the possible association of FLYWCH1 with the cell cycle, we firstly performed PI flow cytometry assays for cell cycle analysis of the KG1a and OCI-AML3 cell lines. The cell lines were selected based on their FLYWCH1 protein expression: the OCI-AML3 cell line had the highest expression and the KG1a cell line had the lowest expression (Figure 2). The flow cytometry and Propidium iodide (PI) staining analysis showed a significant difference in the percentage of cells in the sub-G0 and G0/G1 phases between OCI-AML3 and KG1a cells (Supplementary Figure S3A,B). The KG1a cells had a prolonged sub-G0 phase, while the OCI-AML3 cells had a very short sub-G0 phase with a small number of cells. In contrast, the percentage of cells in G0/G1 transition phase was higher in the OCI-AML3 cell line. The S and G2/M phases were markedly reduced and there was no significant difference between the two cell lines (Supplementary Figure S3A,B).

**Figure 4:**
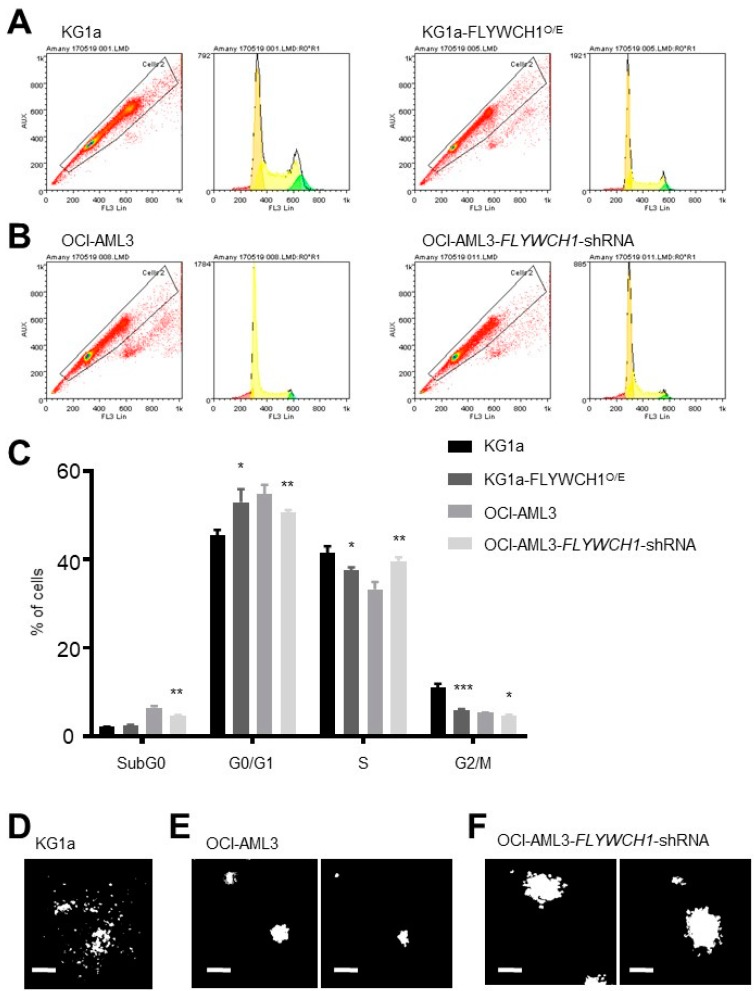
FLYWCH1 plays a role in AML cell growth. (**A**,**B**) Panels show the representatives of flow cytometry and Propidium iodide (PI) staining analysis of (**A**) KG1a control and FLYWCH1 overexpressing, and (**B**) OCI-AML3 control and *FLYWCH1*-shRNMA expressing cell lines. (**C**) The cell cycle status of KG1a and OCI-AML3 cells was monitored via flow cytometry and PI staining in triplicates. Error bars present mean ± SEM of three independent experiments (* *p* < 0.05; ** *p* < 0.01; *** *p* < 0.001). (**D**–**F**) Representative images of (**D**) scattered cell aggregates of KG1a cells and (**E**) colonies from OCI-AML3 control, and (**F**) *FLYWCH1*-shRNMA expressing cell lines. Images were captured at ×10 magnification.

Then, shRNAs targeting the human *FLYWCH1* gene in OCI-AML3 and the overexpression of FLYWCH1 in KG1 were used to down-regulate and up-regulate *FLYWCH1* expression, respectively (Supplementary Figures S2A and S3B). The flow cytometry and PI assays showed that overexpression of FLYWCH1 increased number of cells in G0/G1 arrest, while it decreased the number of cells at S and G2/M transitions in KG1a cells. In contrast, FLYWCH1-knockdown in OCI-AML3 cells decreased the number of cells at G0/G1 transition and increased the number of cells in S phase transition (Figure 4A–C). Therefore, taken together, all these results demonstrate that the expression levels of FLYWCH1 may influence cell proliferation in AML cells.

### 2.5. Low FLYWCH1-Expressing KG1a Cells Formed Scattered Colonies in Comparison with Typical Colony Formation in the High FLYWCH1-Expressing OCI-AML3 Cells

Further functional analysis of stemness activity in vitro was based on the ability of OCI-AML3 and KG1a cell lines for growth and establishment of hematopoietic/progenitor-like cells in colony forming unit (CFU) assays using MethoCult H4230 methylcellulose-based medium [35]. However, the KG1a cells formed some scattered multicellular aggregates but did not organize as common and colony units (Figure 4D). To confirm FLYWCH1 mediated in regulating of the stemness activity, the CFU assay was further examined in KG1a and OCI-AML3 cell lines with, and without, modulation of FLYWCH1 expression as outlined in 2.4. The data showed that scattered cell aggregates morphology of KG1 cell-colonies with FLYWCH1 overexpression remains mostly unchanged (Figure 4D), while FLYWCH1-knockdown in OCI-AML3 cells increased the size of colony-forming units (96 ± 4 μm vs. 158.5 ± 8.5 μm, *n* = 16) (Figure 4E versus Figure 4F), but not significantly the number of CFUs (4.2 versus 4.5)%, when compared to parental cells.

### 2.6. The c-JUN and c-MYC Expressed at Higher Levels in Comparison to FLYWCH1 in Primary AML Samples

**Figure 5:**
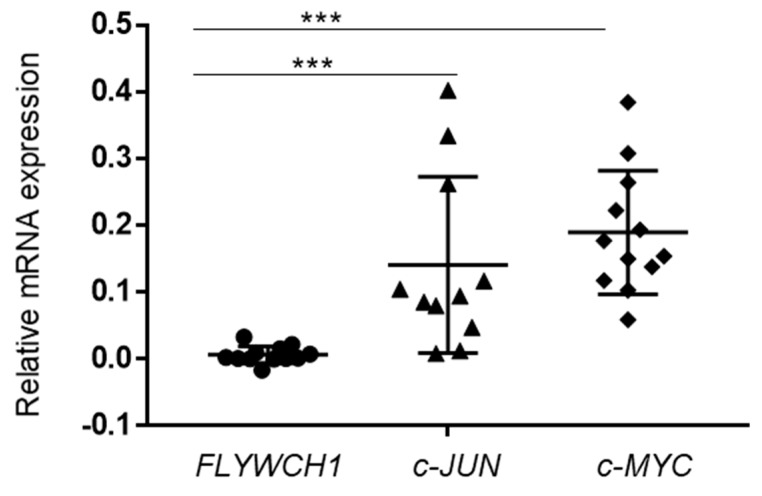
Comparison of *FLYWCH1*, *c-MYC*, and *c-JUN* mRNA expression in AML primary samples. A qRT-PCR analysis of *FLYWCH1*, *c-JUN*, and *c-MYC* mRNA expression in a cohort of twelve AML samples. Data are mean ± SD (*n* = 3; *** *p* < 0.001).

We have examined the expression level of *FLYWCH1* mRNA and compared with the expressions of *c-MYC* and *c-JUN* proto-oncogenes in human primary AML samples. Notably, qRT-PCR analysis of twelve cases indicated a relatively low expression of *FLYWCH1* mRNA in all AML patients (Figure 5). These findings were irrespective of the genetic background of the AML samples. In addition, recent deep sequencing studies indicate that 94% of all protein-coding genes generate multiple mRNA transcripts [36]. Our results in Section 2.1 and Section 2.2 also showed a dissociation between FLYWCH1 mRNA and protein levels in cell lines. Hence, further studies require exploring the possible correlations at the FLYWCH1 mRNA and/or protein level with β-catenin target genes-committed in AML patients. 

## 3. Discussion

It is well-known that aberrant activation of the canonical Wnt signalling pathway (or Wnt/β-catenin pathway) promotes tumour progression and initiation. Recent data suggest that Wnt/β-catenin signalling is linked with AML pathogenesis [10,13,37,38]. The β-catenin plays a very important role in this pathway; therefore, the regulation of β-catenin activity is crucial. Several processes, such as ligand–receptor interactions, nuclear translocation, and transcriptional regulators (repressors and activators), regulate the Wnt/β-catenin pathway [39,40]. The ability to control β-catenin protein is linked to tight regulation of Wnt-mediated signalling, which is necessary for regulating different types of cancer cells, including AML cells. However, the mechanism(s) that control β-catenin are still unclear. Our data suggest that FLYWCH1, a novel protein, is involved in this regulatory process in AML.

Our results suggest that there is differential expression of FLYWCH1 at both transcriptional and translational level. However, the AML cell lines expressed different levels of FLYWCH1 mRNA and protein (Figure 1 and Figure 2). This difference could be associated with the differing biological phenotype of each cell line. For example, Saland et al. [41] suggested that the KG1a cell line has a less aggressive phenotype in comparison with the other AML cell lines, which may be due to it expressing the lowest FLYWCH1 levels (Figure 2, arrowheads); however, this requires further investigation [41]. The difference in FLYWCH1 expression appears to be strongly correlated with the expression of downstream targets of the Wnt/β-catenin pathway. To examine this possible association, we investigated the expression of the downstream proteins, i.e., cyclin D1, c-Myc, and c-Jun, as they are transcriptionally regulated by β-catenin and are implicated in cell cycle control and tumour progression [42]. Cyclin D1, c-Myc, and c-Jun expression were repressed in the OCI-AML3 cell line, which had the highest FLYWCH1 expression (Figure 3A). In the KG1a cell line, which had the lowest FLYWCH1 expression, the proteins were highly expressed (Figure 3A). Our data suggest that FLYWCH1 is a negative regulator of the Wnt/β-catenin signalling pathway. This observation requires further investigation, as other factors could be involved in regulating the gene expression in a cell type- and/or gene-specific manner.

In addition, the data suggest that the differential expression of FLYWCH1 correlates with cell cycle phase transition, as we observed the loss of FLYWCH1 expression in dividing cells or in cells immediately after division (Figure 4A,B). The observation of the possible cell cycle dependence on FLYWCH1 expression was a subject for further examination. Cell cycle analysis suggested that there is a prolonged sub-G0 phase for the KG1a cell line alongside a shortened G0/G1 phase. The sub-G0 phase usually determines apoptotic and necrotic cell death, although we observed an increase in the sub-G0 population in the low FLYWCH1 expressing KG1a cell line. In the OCI-AML3 cell line, however, which showed significantly higher number of cells in G0/G1, there was only a low, almost non-existent sub-G0 phase. These data suggest that FLYWCH1 expression promotes G0/G1 transition and G1 arrest.

Further functional analysis of FLYWCH1 was performed through the stemness activity assay. The OCI-AML3 cells had low colony formation efficiency, whereas the KG1a cells were notable for not forming colonies at all (Figure 4C). Famili et al. [43] reported that higher Wnt/β-catenin pathway activation results in reduced stemness activity in HSCs due to *APC* mutation. In contrast, other investigators have suggested that LSCs tolerate higher Wnt levels [43]. However, higher levels of Wnt in LSCs may be correlated with a more inactive form of LSCs. One question that warrants further investigation is why AML cell lines with higher G0/G1 arrest are usually related with a more quiescent phenotype, showing higher stemness activity. A possible answer may be that the colony formation assay does not necessarily demonstrate stemness activity but its growing potential. The assay has its limitations, as it is not always suitable for detecting more immature progenitor cells or stem cells [44]. Such cells usually require more time to form colonies and, therefore, a long-term culture-initiating cell assay might be more appropriate [44,45].

Mechanistic understanding of the link between these effects and FLYWCH1/β-catenin require further investigation. We speculate that the observed differences in different levels of growth activator are related to oncogenes as their target genes. For example, c-Myc is a proto-oncogene that is correlated with the control of cellular proliferation, stemness, and programmed cell death [46,47]. Karn et al. have suggested that c-Myc overexpression results in a shortened G0/G1 phase [48,49]. Conversely, cyclin D1 plays an active role in the cell cycle, controlling G1/S transition; cyclin D1 overexpression in epithelial cells triggers apoptosis [50]. However, cyclin D1 overexpression appears to be cell type-dependent. In addition, active β-catenin via c-Jun decreases cell adhesion properties, thereby suppressing exposure to survival signals and increasing apoptosis. These data agree with our findings, whereby FLYWCH1, which suppresses c-Myc, c-Jun, and cyclin D1, correlated positively with higher G0/G1 arrest and correlated negatively with apoptosis. However, further investigation is needed to confirm this observation [51,52]. To confirm the correlation of cell cycle control and FLYWCH1, cell cycle analysis after double thymidine synchronization in G1/S and subsequent staining with FLYWCH1 could be used to quantify the protein levels in specific phases of the cell cycle.

Many laboratories are examining potential inhibition strategies for Wnt/β-catenin signalling in a variety of diseases, including cancer. However, despite these efforts, therapeutic agents specifically targeting the Wnt pathway have not been introduced in the clinic. In the present study, we identify for the first time FLYWCH1 as a potential negative regulator of the downstream targets of the Wnt/β-catenin pathway in AML cells. Hence, FLYWCH1 might be an intrinsic inhibitory factor of the oncogenic nuclear β-catenin in AML cells.

## 4. Material and Methods

### 4.1. Cell Culture

The MOLM-13, M0-7e, and OCI-AML3 myeloid leukaemia cell lines were obtained from the German Collection of Microorganisms and Cell Cultures (DSMZ, Braunschweig, Germany). U937, HL-60, and KG-1a cell lines were from the European Collection of Animal Cell Cultures (Salisbury, UK). The MV4.11 and TF1A cell lines were from the American Tissue Culture Collection (Manassas, VA, USA). M0-91 were a kind gift from Dr. Joseph M. Scandura (Sloan-Kettering Institute, USA) [32]. The human AML cell lines M091, U937, M07e, MOLM-13, OCI-AML3, KG1a, MV4-11, TF-1a, and HL-60 were cultured in RPMI 1640 medium (Sigma-Aldrich, Gillingham, UK, R0883) supplemented with 10% fetal bovine serum (FBS, Sigma-Aldrich, Gillingham, UK, F7524), 2 mM L-glutamine (Thermo Fisher Scientific, Loughborough, UK, 25030-024), and 1% penicillin/streptomycin (100 units/mL, Invitrogen, Loughborough, UK, 15140-122). The M07e and KG1a cell lines were cultured as above, but supplemented with 20% fetal calf serum and 10 ng/mL granulocyte-macrophage colony–stimulating factor (Millipore, Watford, UK, GF004). All experiments were performed when cells were in the stationary phase. 

### 4.2. RNA Isolation, Reverse Transcription (RT), and Quantitative Real-Time PCR (qRT-PCR)

Total RNA was extracted from the AML cell lines using TRIzol (Sigma-Aldrich, Gillingham, UK, T9424) according to the manufacturer’s instructions. Total RNA (2 µg) was used to synthesize complementary DNA using a PrimeScript RT reagent kit (Takara-Clontech Laboratories, ST. Germain-en-Laye, France, RR037A) following the manufacturer’s instructions. *Taq* polymerase (Promega, Southampton, UK, 9PIM300) and SYBR Green master mix (Roche, Welwyn Garden City, UK 04707516001) were used to perform PCR and quantitative RT-PCR (qRT-PCR), respectively. The comparative CT method was applied for quantification of gene expression. β-actin was used as endogenous controls, unless otherwise stated. The following primers were used:

*FLYWCH1*-Fwd: 5′-CTGGATGCAGCCCCTCAGT-3’ 

*FLYWCH1*-Rev: 5-′TTGGCGGCACTTCCAGTAC-3’

*β-actin*-Fwd: 5′-GCGCGGCTACAGCTTCA-3’

*β-actin*-Rev: 5′-CTTAATGTCACGCACGATTTCC-3’

*CXXC4-*FWD*: 5’-*ATGCACCACCGAAACGACTC-3’

*CXXC4-*Rev: 5’-GCAGTGTTCAGGGGATAAGGT-3’

*FGF-4*-FWD*: 5’-*TATGGCTCGCCCTTCTTCAC-3’

*FGF-4*-Rev: 5′-TCGGTTCCCCTTCTTGGTCTTC-3’

*PPARD*-Fwd: 5′-AAGAGGAGGAGAAAGAGGAAG-3’

*PPARD*-Rev: 5′-TGAACACCGTAGTGGAAGC-3’

*c-Jun*-Fwd: 5′-ACCCCAAGATCCTGAAACAG-3’

*c-Jun*-Rev: 5′-ATCAGGCGCTCCAGCTCG-3’

*LGR5*-Fwd: 5′-GACAACAGCAGTATGGACG-3’

*LGR5*-Rev: 5′-GCATTACAAGTAAGTGCCAG-3’

C-MYC-Fwd: 5′-GGAGACACCGCCCACCA-3’

C-MYC-Rev: 5′-GCGCTGCGTAGTTGTGCTG-3’

### 4.3. Western Blotting

Radio-immunoprecipitation assay (RIPA) buffer (Sigma-Aldrich, Gillingham, UK, R0278) freshly supplemented with a 1:100 cocktail of phosphatase and protease inhibitors (Sigma-Aldrich, Gillingham, UK, P8340) was used for protein extraction. The protein was quantified using the Bradford assay (Sigma-Aldrich, Gillingham, UK, B-6916). Equal amounts of protein were separated using sodium dodecyl sulfate–polyacrylamide gel electrophoresis (SDS-PAGE), and the separated proteins were transferred to a hydrophobic polyvinylidene difluoride membrane (Fisher Scientific, Loughborough, UK, RPN303F). Blots were blocked using 5% bovine serum albumin (BSA) in Tris-buffered saline (TBS) and 0.1% Tween-20 (TBS-T, Sigma-Aldrich, Gillingham, UK, P1379) for 1 hour at room temperature. Then, the blots were incubated at 4 °C with anti-primary antibody in 5% BSA–TBS-T overnight. The antibodies used were those against: cyclin D1 (Santa Cruz, Heidelberg, Germany, H295 SC-753, 1:500 dilution), c-Myc (Thermo Fisher Scientific, Loughborough, UK, MA 1-980 9e10, 1:500 dilution), c-Jun (Santa Cruz, Heidelberg, Germany, H79 Sc1694, 1:500 dilution), and β-actin (Santa Cruz, Heidelberg, Germany, Sc47778 C4, 1:5000 dilution). To detect the protein bands, fluorescent secondary antibodies (1: 5000 dilution) were measured using an Odyssey Fc Dual-Mode Imaging System (LI-COR Biosciences).

### 4.4. Immunofluorescence Assay

Immunofluorescence was carried out following standard procedures [22]. Briefly, cells (3 × 10^4^) suspended in 100 µL of phosphate-buffered saline (PBS) were plated on slides after cytospin at 800 rpm for 3 mins (Thermo Fisher Scientific). The cells were fixed in 4% paraformaldehyde, blocked with 3% BSA, and washed in 3% BSA and 0.1% phosphate-buffered saline (PBS)–Tween-20. The cells were probed overnight at 4 °C with primary antibody targeting FLYWCH1 (Santa Cruz, Heidelberg, Germany, v-18 Sc242835, 1:50 dilution), followed by secondary Alexa flour labelled antibody (Life Technologies, Loughborough, UK, A11057, 1:500 dilution) and DAPI (4′, 6-diamidino-2-phenylindole) staining, and mounted with ProLong Diamond Antifade Mountant with DAPI (Invitrogen, Loughborough, UK, P36962). Images were captured using a Leica fluorescent microscope.

### 4.5. Plasmids

The IMAGE-clone of human FLYWCH1-cDNA purchased from Geneservice (ID: 4839062/AK34; GenBank: 84256). The human *FLYWCH1* gene is composed of 2151 nucleotides encoding a protein with 716aa residues called FLYWCH1. The IMAGE-clone plasmid DNA then digested with several restriction-enzymes and cloned into different vectors, while fused with the eGFP in pLVX-Puro backbone vector, as previously described [22]. The scrambled piLenti-scrambled control shRNA (scs)-GFP, and piLenti-FLYWCH1-set-shRNA-GFP (set of 4) from ABM Inc. (https://www.abmgood.com/FLYWCH1-shRNA-Lentivectors-i008000.html) as previously described [22]. 

### 4.6. Lentivirus Production and Cell Transduction

Human EK293T cells maintained in RPMI supplemented with 10% FBS. Cells transiently transfected using Lipofectamine-2000 (Invitrogen, Loughborough, UK, 11668027) and processed for virus production, as described [22]. Knocked-down for FLYWCH1 by lentiviral transduction of piLenti-FLYWCH1-set-shRNA-GFP and control piLenti-scsRNA (scrambled control shRNA)-GFP. Both cells selected in a Puromycin (Sigma-Aldrich, Gillingham, UK, P8833)-containing medium for 2 weeks then the resistant cells further amplified and expanded. The knocked-down status of FLYWCH1 was validated through RT-PCR.

### 4.7. Cell Cycle Analysis

The AML cells were washed in cold PBS, fixed in ice-cold 70% ethanol, and stained for 15 min at room temperature with propidium iodide (PI, Sigma-Aldrich, Gillingham, UK, 4864) and RNase (Sigma-Aldrich, Gillingham, UK, 6513) [21]. The cell cycle analysis was performed using flow cytometry (FC500 flow cytometer, Beckman-Coulter). All samples were loaded in triplicate.

### 4.8. Colony Formation Assay

Cells (1 × 10^3^) were seeded in triplicate in 24-well plates in a semi-solid matrix (MethoCult H4230 [an incomplete methylcellulose-based medium], STEMCELL Technologies, Cambridge, UK, 04236) and incubated for 14 days according to the manufacturer’s protocol. The colonies were monitored microscopically every 2 days, identified and counted on day 14, and images were captured using a phase-contrast microscope.

### 4.9. Patient Primary Samples

Blood or bone marrow samples were obtained at diagnosis after written consent from adult patients with AML (excluding M3 patients). National and Local Research Ethics Committees approved the use of these samples (Ethics reference number—06/Q2403/16, Date 19 November 2018, East Midlands Committee—Nottingham 1 REC). Mononuclear cells were isolated using a standard density gradient centrifugation method with histopaque and were cryopreserved in liquid nitrogen. RNA was prepared from AML blasts using QIAamp RNA kits with DNase treatment according to the manufacturers’ instructions (Qiagen, UK, 52304). Up to 2 μg of RNA was used in a reverse transcription reaction with MMLV reverse transcriptase (Invitrogen, Paisley, UK, 28025013) and random primers (Invitrogen, Paisley, UK, 48190011).

### 4.10. Statistical Analyses

Statistical analyses were performed by the two-tailed Student’s *t*-test using Microsoft Excel. Significance testing was performed using SPSS version 15. Mean ± SD (* *p* < 0.05; ** *p* < 0.01; *** *p* < 0.001) values are shown.

## 5. Conclusions

Differential expression of FLYWCH1 appears to be an important determining factor to balance the β-catenin/Tcf activity within AML cells. There seem to be at least two different stages at which dysregulation of Wnt/β-catenin signalling plays a role in leukemogenesis. First, at the initiation phase of the disease, elevated Wnt signalling could be the crucial factor to progress from a pre-LSC into an LSC (as for AML), as has been recently discovered for T-ALL (T-cell acute lymphoblastic leukaemia) where the absence of the Wnt-nuclear factor Tcf1 appears to predispose to T-ALL development. However, for CML (chronic myeloid leukaemia), ectopically activated Wnt signalling is also essential. Hence, depending on the differentiation status of the cell and or the location of the leukaemia-initiating cells in its microenvironment, Wnt/β-catenin signalling plays different roles in leukemogenesis. Thus, a dysregulated FLYWCH1/β-catenin signalling can contribute to leukemogenesis, both on the differentiation and leukemic status of the cell. The data warrant further investigation of FLYWCH1/β-catenin signalling as a critical component in all hematologic malignancies including AML pathogenesis.

## Supplementary Materials

Supplementary materials can be found at https://www.mdpi.com/1422-0067/20/11/2739/s1.

## Abbreviations

AML	Acute myeloid leukaemia
CFU	Colony forming unit
CML	Chronic myeloid leukaemia
CSCs	Cancer stem cells
FACS	Fluorescence activated cell sorting
FLYWCH1	FLYWCH-type zinc finger 1
GADPH	Glyceraldehyde 3-phosphate dehydrogenase
HSC	Hematopoietic stem cell
LSCs	Leukaemia stem cells
mRNA	Messenger RNA
qPCR	Quantitative real-time polymerase chain reaction
PI	Propidium iodide
RIPA	Radio-immunoprecipitation assay
RNA	Ribonucleic acid
RRS	Ras-Recruitment System
scsRNA	Scrambled control shRNA
shRNA	small hairpin RNA
T-ALL	T-cell acute lymphoblastic leukaemia
ZNF	Zinc Finger Protein
