# Supplementary material for: Increased FLYWCH1 Expression is Negatively Correlated with Wnt/β-catenin Target Gene Expression in Acute Myeloid Leukemia Cells
Source: Int J Mol Sci. 2019 Jun 4;20(11):2739. doi: 10.3390/ijms20112739 (PMC6600431; doi:10.3390/ijms20112739)
Supplement: Supplementary file 1 [file ijms-20-02739-s001.pdf]

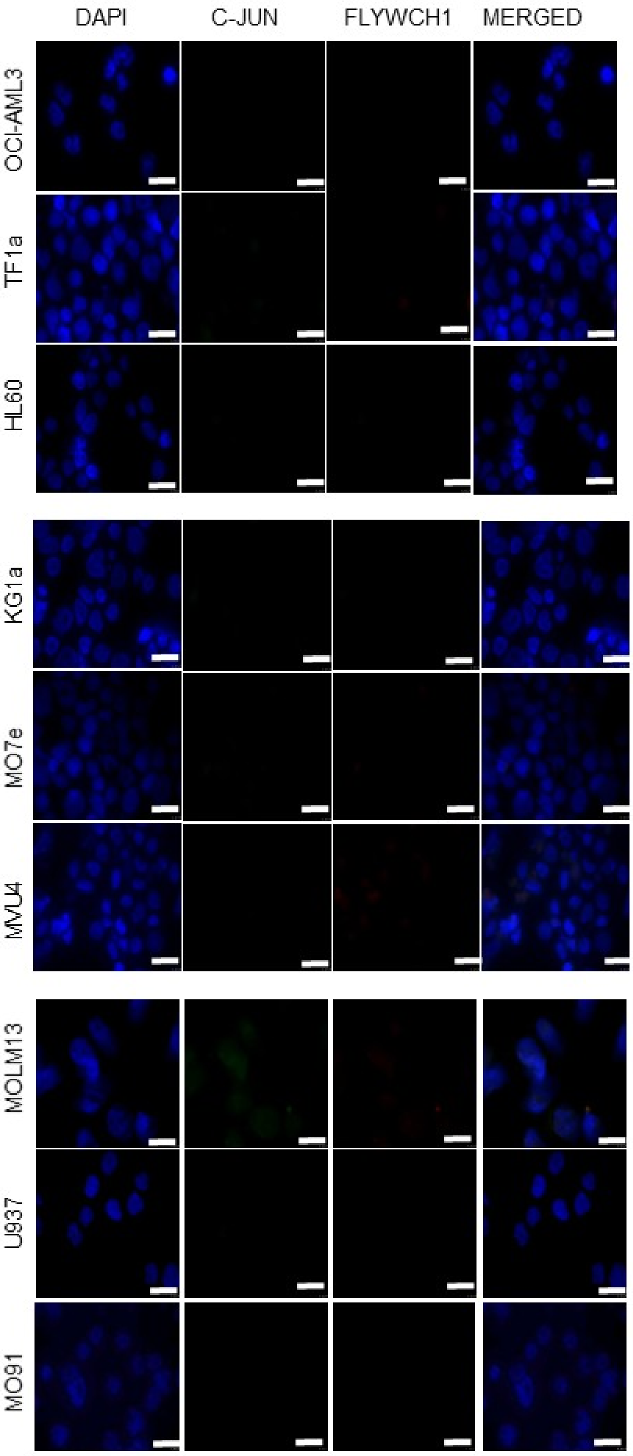

Supplementary
Figure 1, Almars A, et al.

Supplementary Figure 1. Images are the control cells stained for the secondary antibody only. The secondary antibodies showed no background in the negative controls, however there was a very little background in fluorescent images for MVU4 and MOLM13 cells. Scale bars, 50 μm.

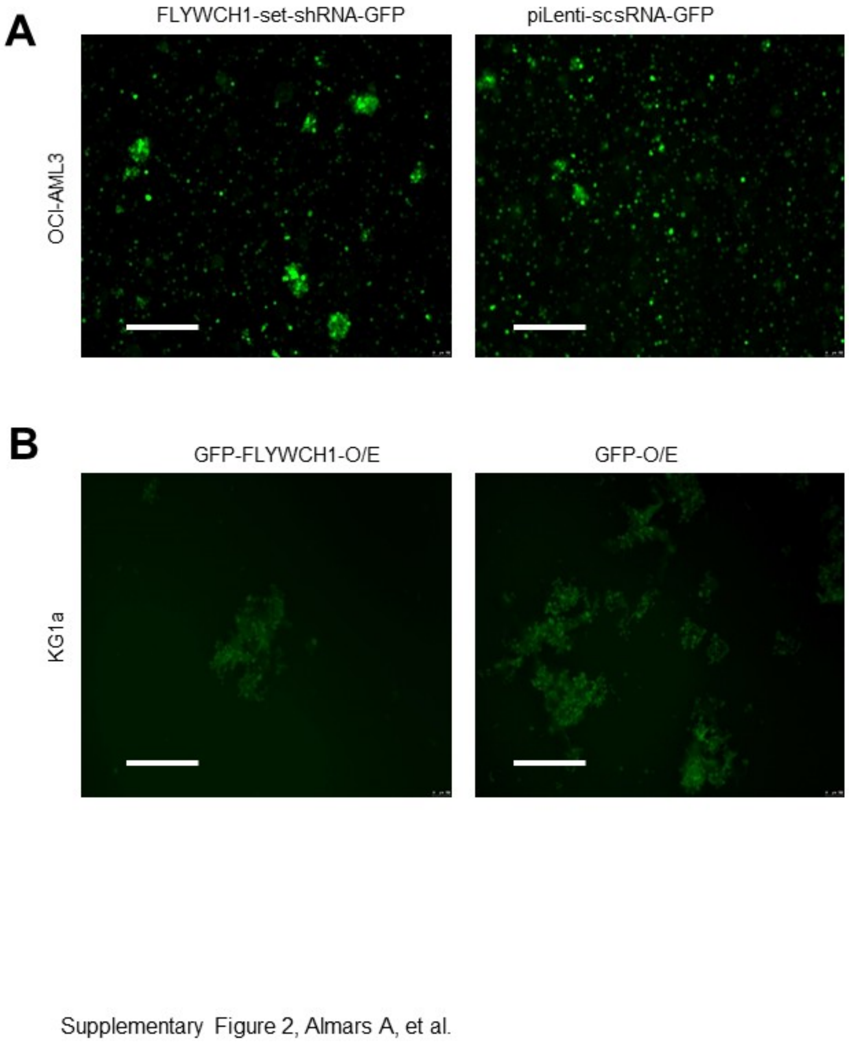

Supplementary Figure 2. A) Representative fluorescent images of OCI-AML3 cells transduced with GFP-expressing lentiviral vectors; FLYWCH1-set-shRNA-GFP (left panel), piLenti-scsRNA (scrambled control shRNA) –GFP (right panel). **B)** Representative fluorescent images of KG1a cells transduced with GFP-expressing lentiviral vectors expressing the full-length cDNA of human FLYWCH1 tagged with eGFP (GFP-FLYWCH1) and pLV-GFP backbone vector. Scale bars, 100 μm.

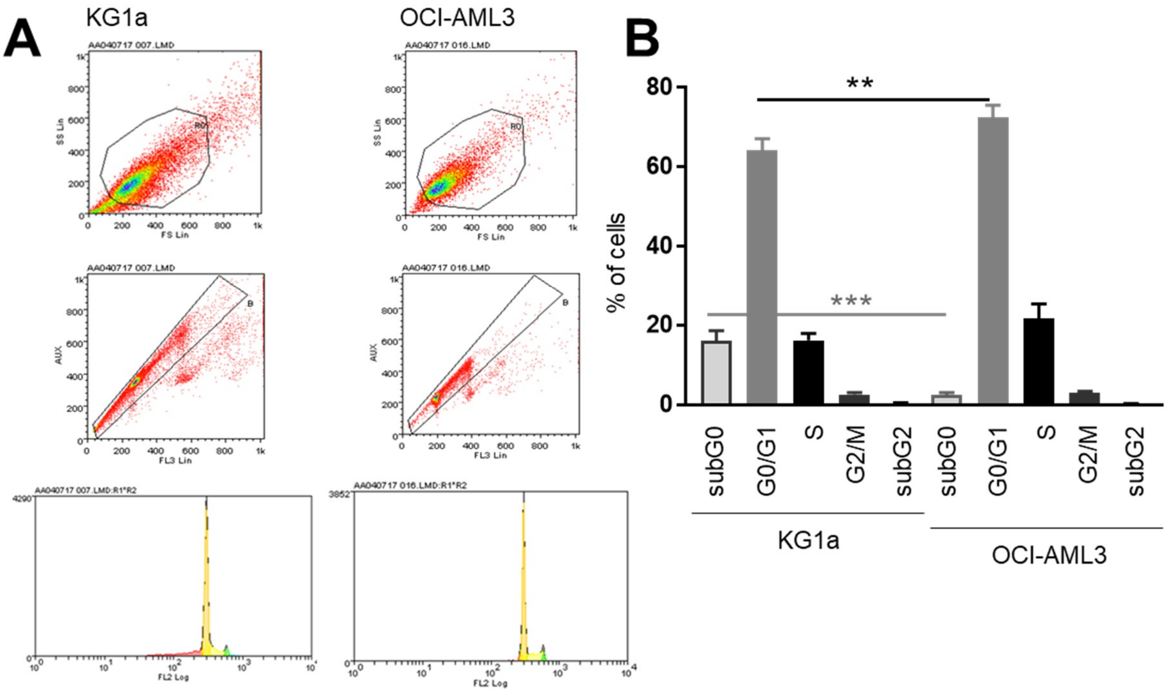

Supplementary Figure 3, Almars A, et al.

Supplementary Figure 3. A) Panels show the representatives of flow cytometry and Propidium iodide (PI) staining analysis of KG1a and OCI-AML3 cell lines. **B)** The cell cycle status of KG1a and OCI-AML3 cells was monitored via flow cytometry and PI staining in triplicates. Error bars present mean ± SEM of three independent experiments (*, $P < 0.05$; **, $P < 0.01$; ***$P < 0.001$).
